# Structural and Functional Brain Correlates of Cognitive Impairment in Euthymic Patients with Bipolar Disorder

**DOI:** 10.1371/journal.pone.0158867

**Published:** 2016-07-22

**Authors:** Silvia Alonso-Lana, José M. Goikolea, Caterina M. Bonnin, Salvador Sarró, Barbara Segura, Benedikt L. Amann, Gemma C. Monté, Noemi Moro, Paloma Fernandez-Corcuera, Teresa Maristany, Raymond Salvador, Eduard Vieta, Edith Pomarol-Clotet, Peter J. McKenna

**Affiliations:** 1 FIDMAG Germanes Hospitalàries Research Foundation, Barcelona, Spain; 2 Centro de Investigación Biomédica en Red de Salud Mental (CIBERSAM), Madrid, Spain; 3 Programa de Doctorado de Medicina, University of Barcelona, Barcelona, Spain; 4 Bipolar Disorder Program, Institute of Neuroscience, Hospital Clínic, University of Barcelona, IDIBAPS, Barcelona, Catalonia, Spain; 5 Department of Psychiatry and Clinical Psychobiology, University of Barcelona, Barcelona, Spain; 6 Benito Menni Complex Assistencial en Salut Mental, Barcelona, Spain; 7 Hospital Sant Joan de Déu Infantil, Barcelona, Spain; UTHSCSH, UNITED STATES

## Abstract

**Introduction:**

Cognitive impairment in the euthymic phase is a well-established finding in bipolar disorder. However, its brain structural and/or functional correlates are uncertain.

**Methods:**

Thirty-three euthymic bipolar patients with preserved memory and executive function and 28 euthymic bipolar patients with significant memory and/or executive impairment, as defined using two test batteries, the Rivermead Behavioural Memory Test (RBMT) and the Behavioural Assessment of the Dysexecutive Syndrome (BADS), plus 28 healthy controls underwent structural MRI using voxel-based morphometry (VBM). Twenty-seven of the cognitively preserved patients, 23 of the cognitively impaired patients and 28 controls also underwent fMRI during performance of the n-back working memory task.

**Results:**

No clusters of grey or white matter volume difference were found between the two patient groups. During n-back performance, the cognitively impaired patients showed hypoactivation compared to the cognitively preserved patients in a circumscribed region in the right dorsolateral prefrontal cortex. Both patient groups showed failure of de-activation in the medial frontal cortex compared to the healthy controls.

**Conclusions:**

Cognitive impairment in euthymic bipolar patients appears from this study to be unrelated to structural brain abnormality, but there was some evidence for an association with altered prefrontal function.

## Introduction

Studies over the last two decades have demonstrated that a proportion of patients with bipolar disorder show cognitive impairment that persists beyond episodes of illness into euthymia [1]. The deficits are wide ranging [2], but may involve executive function and long-term memory particularly [1], and they are associated with impaired functioning in daily life [3, 4]. Presence of residual mood disturbance does not appear to fully account for the impairment seen [5], nor, according to a meta-analysis, does treatment with antipsychotic drugs [6]. Lithium [7] and anticonvulsants [8] have been found to impair only some areas of cognitive function in bipolar patients and so also appear to be unlikely to be the whole explanation.

Since patients with bipolar disorder do not show evidence of premorbid intellectual disadvantage [9–11], some form of brain dysfunction presumably underlies this form of persistent cognitive impairment. One possibility is that it is a consequence of structural brain pathology. Bipolar disorder is known to be associated with lateral ventricular enlargement [12–14], and there is evidence for a small reduction in brain size, although this reached significance in only one of two meta-analyses [13, 14]. Studies using whole-brain techniques such as voxel-based morphometry (VBM) have additionally found evidence for volume reductions in the anterior cingulate cortex, the insula and the inferior frontal cortex, among other regions [15–18]. White matter changes are also well documented in bipolar disorder, both in the form of subcortical signal hyperintensities [19] and reduced fractional anisotropy on diffusion tensor imaging (DTI); the latter changes have been found most consistently in the right temporo-parietal and the left anterior and mid-cingulate regions [20].

Relatively few studies have examined whether structural changes in bipolar patients are related to presence of cognitive impairment. Early studies reviewed by Bearden et al [21] found some evidence of associations with increased lateral ventricular volume and volume reductions in the prefrontal cortex, and more robustly with presence of white matter signal intensities. However, more recent studies examining multiple grey and white matter regions have generally found few significant correlations with executive, memory or other cognitive deficits [22–26].

Findings from many functional imaging studies in bipolar disorder have led to a consensus that it is characterized by reduced resting and task-related activity in the prefrontal cortex and some other cortical regions, coupled with overactivity in the amygdala, hippocampus and parahippocampal gyrus and the basal ganglia [27]. Not all of these abnormalities are seen in euthymia, however. Thus, in a meta-analysis pooling effect size data from PET, SPECT and fMRI studies, Kupferschmidt et al [28] found that euthymic patients showed evidence of task-related hypoactivations in the inferior and middle frontal cortex and the dorsolateral prefrontal cortex (DLPFC), as well as hyperactivity in the superior temporal gyrus and ventrolateral prefrontal cortex. On the other hand, in a meta-analysis of voxel-based studies, Chen et al [29] found evidence only for reduced activation in the lingual gyrus in euthymic patients.

To date, very few studies have investigated brain activations in relation to cognitive impairment in bipolar disorder [30–32]. In one study that examined patients in the euthymic phase, Oertel-Knöchel et al [33] found that 26 euthymic bipolar patients were impaired on a verbal learning and recognition task, and also showed a pattern of reduced activation compared to healthy controls when they performed the same task while being scanned. The areas affected included the left middle and superior frontal gyrus during encoding, and the bilateral middle and inferior frontal gyrus, plus the parahippocampal and other posterior medial cortical areas during retrieval.

The aim of this study was to determine whether and to what extent cognitive impairment in euthymic bipolar patients has brain structural and/or functional correlates. To do this, we recruited groups of demographically well-matched patients who either showed or did not show executive and/or memory impairment, defined according to predetermined criteria, in the euthymic phase. Healthy controls were also employed. Both whole-brain structural imaging (VBM) and functional imaging (cognitive task-related fMRI) were carried out.

## Materials and Methods

### Participants

The patient sample consisted of two groups of adults with bipolar disorder, who were prospectively recruited on the basis of showing (N = 28) or not showing (N = 33) cognitive impairment (as defined below) in the euthymic phase. Patients were from the outpatient departments of two psychiatric hospitals in Barcelona: Benito Menni CASM and the University of Barcelona Hospital Clínic. They all met DSM-IV criteria for bipolar I disorder and were required to have had at least two episodes of illness. Patients were excluded if a) they were younger than 18 or older than 55; b) they had a history of brain trauma or neurological disease, c) they had shown alcohol/substance abuse within 12 months prior to participation; d) they had undergone electroconvulsive therapy in the previous 12 months; and e) they showed evidence of general intellectual impairment/handicap, as indexed by a current IQ outside the normal range (i.e. below 70) as measured using four subtests of the Wechsler Adult Intelligence Scale III (WAIS-III) (vocabulary, similarities, block design, and matrix reasoning). All patients were right-handed.

Patients were considered to be euthymic if they had had no episodes of illness for at least three months and if they had a score on Hamilton Rating Scale for Depression (HDRS-21) of ≤ 8 and Young Mania Rating Scale (YMRS) of ≤ 8 at the time of testing. These quite strict requirements were used in order to avoid the potentially confounding effects of subthreshold depressive and manic symptoms on cognitive function [34]. The upper age limit of 55 was chosen in order to exclude late-onset affective disorder which has an association with vascular and neurodegenerative disease and so might be independently associated with cognitive impairment [35].

Patients in the cognitively preserved group were on treatment with mood stabilizers (lithium alone n = 13, other mood stabilizers alone n = 6; lithium in combination with other mood stabilizers n = 9), antidepressants (n = 8) and antipsychotics (n = 21; second generation n = 21, first generation n = 2; mean chlorpromazine equivalent dose 284.65 ±337.31 mg/day). The cognitively impaired patients were also on treatment with mood stabilizers (lithium alone n = 13, other mood stabilizers alone n = 4; lithium in combination with other stabilizers n = 7), antidepressants (n = 7); 17 were taking antipsychotics (second generation n = 15, first generation n = 1, both n = 1; mean chlorpromazine equivalent dose 245.20± 209.77 mg/day).

A group of 28 right-handed healthy controls were recruited via poster and web-based advertisement in the hospital and local community, plus word-of-mouth requests from staff in the research unit. The controls met the same exclusion criteria as the patients. They were also excluded if they reported a history of mental illness or treatment with psychotropic medication, and/or had a first-degree relative with a psychiatric illness.

The three groups were selected to be matched for age, sex and estimated IQ (premorbid IQ in the patients). IQ was estimated using the Word Accentuation Test (Test de Acentuación de Palabras, TAP) [36] a pronunciation test that is conceptually similar to the National Adult Reading Test (NART) used in the UK [37] and the Wide Range of Achievement Test (WRAT) in the USA [38]. Subjects have to pronounce low-frequency Spanish words whose accents have been removed. Scores can be converted into IQ estimates [39].

### Cognitive assessment

This was based on Spanish versions of two well-validated memory and executive test batteries, the Rivermead Behavioural Memory Test (RBMT) [40] and the Behavioural Assessment of the Dysexecutive Syndrome (BADS) [41]. These two tests provide a wide ranging assessment of different aspects of memory and executive function, respectively, and are designed to be ‘ecologically valid’, that is to capture the broad range of executive and memory functions required in real-life settings. Both have been subjected to extensive validation in healthy adults and normative data for healthy adults are available.

The RBMT consists of 12 subtests examining verbal recall, recognition, orientation, remembering a route and three measures of prospective memory, the ability to remember to do things. Pass/fail scores are summed to give a ‘screening’ score. The BADS consists of 6 subtests covering cognitive estimation, rule shifting, planning, problem solving and decision making under multiple task demands (the Modified Six Elements Test). Scores from 0 to 4 on each subtest are summed to give an overall ‘profile’ score.

The patients were classified as cognitively preserved or impaired using 5^th^ percentile cutoffs based on normative data for adults. Thresholding for impairment at the 5^th^ percentile for the normal population is an established method in neuropsychology [42]. Specifically, patients were considered cognitively impaired if they scored below the 5th percentile on the RBMT and/or the BADS (screening score of ≤7 on the RBMT and profile score of ≤11 on the BADS), and were considered cognitively preserved if they scored at or above the 5th percentile on both tests (≥8 or more on the RBMT and ≥12 on the BADS).

### Scanning procedure

All subjects underwent structural and functional MRI scanning using a 1.5 Tesla GE Signa scanner (General Electric Medical Systems, Milwaukee, Wis) located at the Sant Joan de Déu Hospital in Barcelona (Spain).

#### Structural neuroimaging

High resolution structural T1-weighted MRI data were acquired with the following acquisition parameters: matrix size 512x512; 180 contiguous axial slices; slice thickness of 1 mm, no slice gap; voxel resolution 0.47x0.47x1 mm^3^; echo time (TE) = 3.93 ms, repetition time (TR) = 2000 ms and inversion time (TI) = 710 ms; flip angle 15°.

Brain structure (grey matter) was examined using FSL-VBM, an optimized VBM style analysis [43, 44] carried out with FSL tools; this yields a measure of difference in local grey matter volume. First, structural images were brain-extracted [45]. Next, tissue-type segmentation was carried out. The resulting grey matter partial volume images were then linearly aligned to MNI 152 standard space [46, 47], followed by nonlinear registration. The resulting images were averaged to create a study-specific template, to which the native grey matter images were then non-linearly re-registered. The registered partial volume images were then modulated by dividing by the Jacobian of the warp field. The modulated gray matter segments were then smoothed with an isotropic Gaussian kernel using a sigma of 4mm (equivalent to Full Width at Half Maximum (FWHM) of 9.4 mm) (technical details are available at www.fmrib.ox.ac.uk/fsl/fslvbm/). Voxel-size after VBM processing was 2x2x2mm.

Group comparisons were performed using permutation-based non-parametric tests. The TFCE (Threshold-Free Cluster Enhancement) method, also implemented in FSL, was used for this purpose. TFCE finds clusters in the data without having to define the initial cluster-forming threshold [48]. Cluster-like structures are enhanced but the image remains fundamentally voxel-wise. In the resulting maps, obtained with 5000 permutations, family-wise error (FWE) rate was used to control for multiple comparisons and only FWE-corrected cluster p-values <0.05 were considered.

We also examined white matter volume. Since the VBM analysis in FSL has only been validated for grey matter, this was carried out with SPM12 (http://www.fil.ion.ucl.ac.uk/spm/software/spm12/). The following standard pre-processing steps were carried out: (1) tissue-type segmentation, (2) normalization to standard space of the obtained white matter images and (3) modulation. The resulting images were then smoothed with an isotropic Gaussian kernel with a sigma of 4 mm. In order to make the results comparable to those reported for grey matter using FSL-VBM, statistical analysis were conducted with the same correction method. That is, all comparisons were carried out with the TFCE method included in FSL, using 5000 permutations and a FWE-corrected threshold of p <0.05.

#### Functional neuroimaging

**Fig 1:**
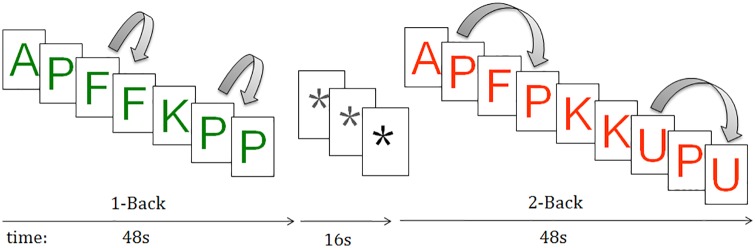
Sequential-letter version of the n-back task with two levels of memory load, 1-back (green) and 2-back (red).

For this we used the n-back task [49], which has been widely employed as a probe for executive function, specifically working memory, in fMRI studies in healthy subjects [50] and psychiatric disorders including schizophrenia [51] and bipolar disorder [52]. Two levels of memory load (1-back and 2-back) were presented in a blocked design manner; in the 1-back task, participants had to respond with a key press when a letter was the same as the one that was presented immediately previously, whereas in the 2-back task they had to respond when the letter was the same as that presented two letters previously (Fig 1). Each block consisted of 24 letters which were shown every two seconds (1 second on, one second off) and all blocks contained five repetitions (1-back and 2-back depending on the block) located randomly within block. Individuals had to detect these repetitions and respond by pressing a button. In order to identify which task had to be performed, characters were shown in green in the 1-back blocks and in red in the 2-back blocks. Four 1-back and four 2-back blocks were presented in an interleaved way, and between them, a baseline stimulus (an asterisk flashing with the same frequency as the letters) was presented for 16 seconds. All individuals went through a training session before entering the scanner.

Performance was measured using the signal detection theory index of sensitivity (d’) of ability to discriminate targets from non-targets [53]. Higher values of d’ indicate better ability to discriminate between targets and distractors. Subjects who had negative d’ values in either the 1-back and 2-back versions of the task, which suggests that they were not performing it, were excluded from the analysis.

In each individual scanning session 266 volumes were acquired. A gradient echo echo-planar sequence depicting the BOLD contrast was used. Each volume contained 16 axial planes acquired with the following parameters: TR = 2000 ms, TE = 20 ms, flip angle = 70 degrees, section thickness = 7 mm, section skip = 0.7 mm, in-plane resolution = 3x3 mm. The first 10 volumes were discarded to avoid T1 saturation effects.

fMRI image analyses were performed with the FEAT module, included in FSL software [54]. Pre-processing with FSL-FEAT included: a) motion correction [47]; b) non-brain removal [45]; c) isotropic 5mm-FWHM Gaussian smoothing; d) high-pass temporal filtering; e) time-series statistical analysis with local autocorrelation correction [55]; and f) registration to the MNI 152 standard space [46, 47]. To minimize unwanted movement-related effects, participants with an estimated maximum absolute movement >3.0 mm or an average absolute movement >0.3 mm were excluded from the study.

General linear models (GLMs) were fitted to generate the individual activation maps for the 1-back vs. baseline, 2-back vs. baseline and 2-back vs. 1-back comparisons. Differences in fMRI activation maps between patients and controls were generated within the FEAT module, using mixed effects GLM models [56]. FEAT uses Gaussian random field theory to properly account for the spatially distributed patterns when performing statistical tests. Specifically, the analyses were performed with the FLAME stage 1 with default height threshold (z > 2.3) [55, 57] and a *p*-value < 0.05 corrected for multiple comparisons [58, 59].

### Ethics statement

All subjects gave written informed consent prior to participation in accordance to the Declaration of Helsinki. Only individuals judged to have decision-making capacity were included. The subjects in the cognitively impaired group were included on the basis that they showed memory and/or executive function as detected during the course of the neuropsychological testing carried out for the purpose of the study, not because they had been found to show clinically significant cognitive impairment by their treating clinicians. The research protocol was approved by the Clinical Research Ethics Committee of the Sisters Hospitallers (Comité de Ética de Investigación Clínica de las Hermanas Hospitalarias), which also approved this method of obtaining informed consent for the study.

### Data analysis

Demographic, clinical and cognitive variables were compared among the groups using SPSS version 17. Normality of continuous variables was examined for and parametric (t-test or ANOVA) or non-parametric tests (Mann-Whitney or Kruskal-Wallis test) were applied as appropriate.

In order to examine the relationship between presence of cognitive impairment and brain structure and function, we carried out two comparisons using a strategy we have employed previously for schizophrenia [60]. First, we contrasted the cognitively preserved group with the control group; this gives a measure of changes in brain structure and/or function that are attributable to bipolar disorder uncontaminated by presence of cognitive impairment. Secondly, to detect changes attributable to the presence of cognitive impairment, we contrasted the cognitively preserved and cognitively impaired patient groups.

## Results

Demographic characteristics of the patients and controls are shown in Table 1. The groups were matched for age, sex and TAP-estimated IQ. There were no differences in psychopathological measures (YMRS and HRSD scores), duration of the illness, functioning (GAF score) and antipsychotic dosage between the cognitively preserved and the cognitively impaired patients (Table 1).

**Table 1:** Demographic, neurocognitive and psychopathological characteristics of the groups.

	Controls (n = 28)	Cognitively preserved (n = 33)	Cognitively impaired (n = 28)	Statistics	Post hoc testing
Age	44.01 (6.03)	44.13 (6.63)	46.17 (7.40)	F = 0.94 p = 0.40	
Sex (male/female)	12/16	18/15	17/11	χ^2^ = 1.85 p = 0.40	
Estimated premorbid IQ (TAP)	105.93 (7.25)	106.03 (6.32)	102.71 (8.81)	H = 3.08 p = 0.21	
BADS profile score	19.18 (2.40)	17.12 (2.25)	13.89 (3.54)	F = 26.09 p<0.001	CI < CP (p<0.001)
					CI < CON (p<0.001)
					CP < CON (p = 0.01)
RBMT screening score	10.61 (1.64)	9.76 (1.41)	6.11 (1.29)	H = 55.43 p<0.001	CI < CP (p<0.001)
					CI < CON (p<0.001)
					CP < CON (p = 0.02)
Duration of illness (years)	-	16.76 (7.44)	19.13 (8.16)	t = 1.17 p = 0.25	
YMRS score	-	1.18 (1.81)	1.77 (2.10)	U = 360.50 p = 0.25	
HRSD score	-	2.55 (2.02)	2.19 (2.35)	U = 367.00 p = 0.33	
GAF score	-	79.07 (11.35)	75.75 (12.72)	t = -0.99 p = 0.33	

Values are given as mean (SD). IQ, intelligence quotient; TAP, Word Accentuation Test; BADS, Behavioural Assessment of the Dysexecutive Syndrome; RBMT, Rivermead Behavioural Memory Test; YMRS, Young Mania Rating Scale; HRSD, Hamilton Rating Scale for Depression; GAF, Global Assessment of Functioning; F, one-way ANOVA test; χ^2^, Chi-square test; H, one-way Kruskal-Wallis test; U, Mann-Whitney test; CON, controls; CP, cognitively preserved; CI, cognitively impaired.

**Fig 2:**
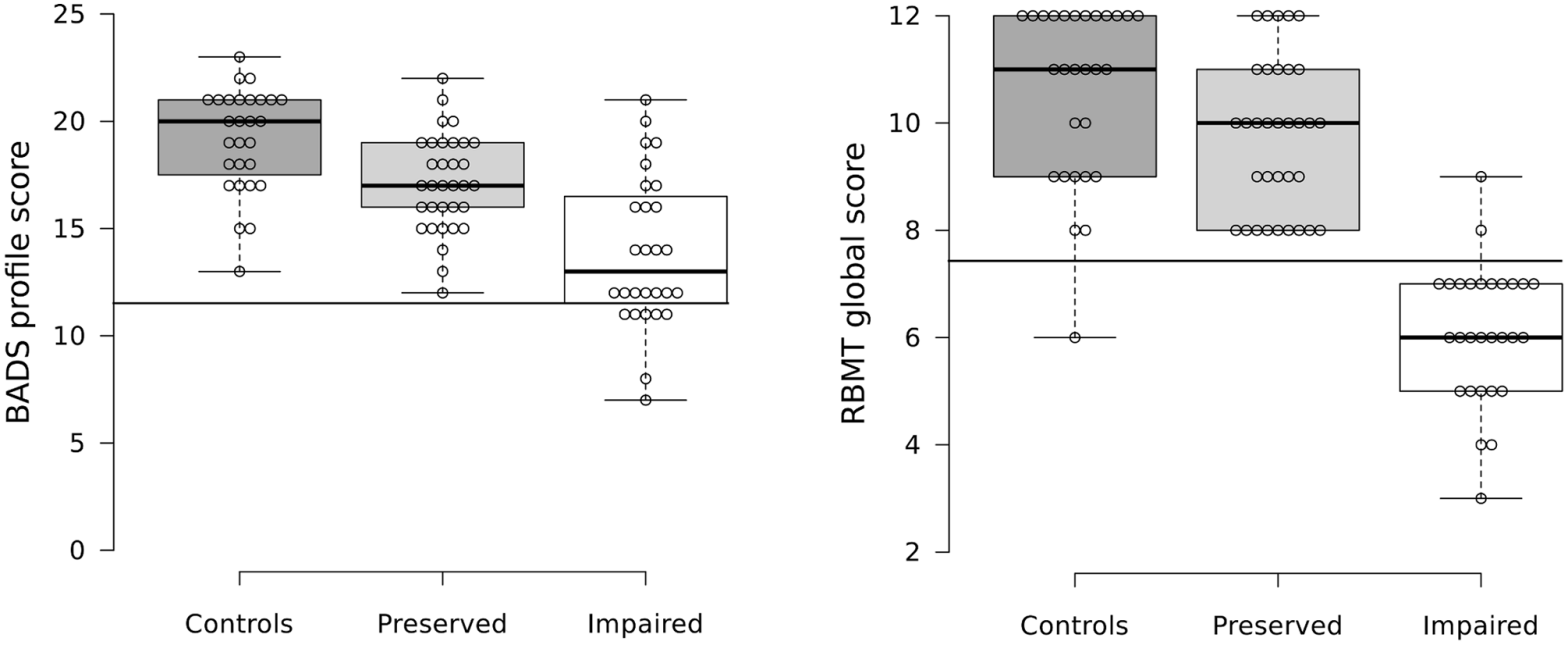
Scatter plots for the controls, cognitively preserved and cognitively impaired groups. Scatter plot of scores on (A) the RBMT and (B) the BADS. The horizontal lines show 5^th^ percentile cutoffs for impairment.

As expected, the two patient groups differed in their performance on the BADS and RBMT (Table 1). The cognitively preserved group was also found to show significant differences from the healthy controls. A scatter plot of scores for all three subject groups is shown in Fig 2 and indicates that this latter finding was due to more cognitively preserved patients falling into low average ranges than the healthy controls.

### VBM findings

#### Controls vs. cognitively preserved patients

**Fig 3:**
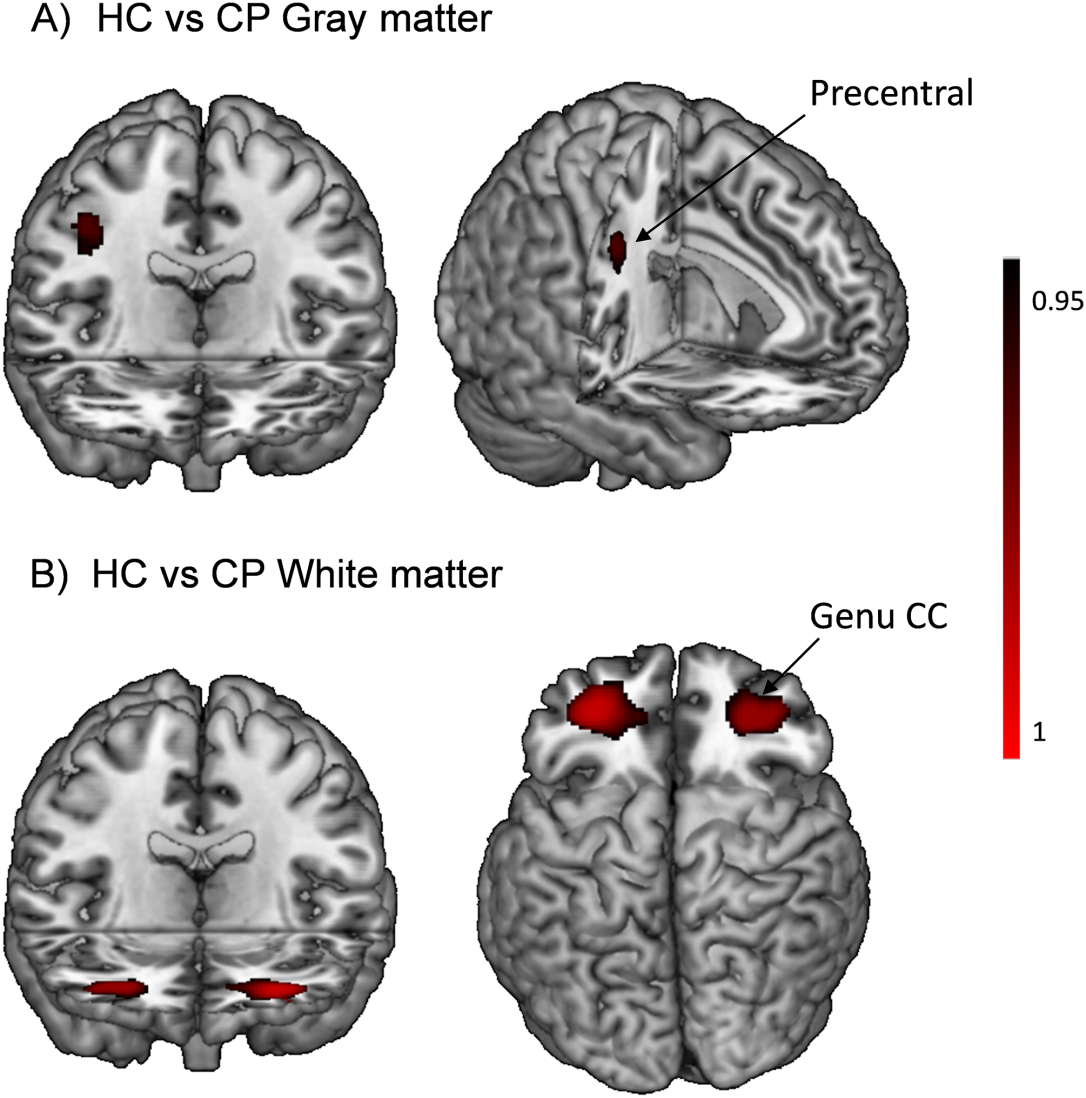
Brain regions showing significant gray and white matter volume reduction in the cognitively preserved patients with bipolar disorder compared with controls.

At p <0.05 corrected, the cognitively preserved patients showed significantly reduced grey matter volume in a single small cluster located in the right precentral gyrus [173 voxels, p = 0.03; peak in BA6, MNI (38,-10,38)] (Fig 3).

The cognitively preserved patients also showed bilaterally symmetrical clusters of significantly reduced white matter volume compared to the controls. On the left side, a cluster extended from the inferior occipito-frontal and uncinate fasciculus to the genu of corpus callosum [2966 voxels, p = 0.01, peak in MNI (-30, 46.5, 1.5)]. A second smaller cluster on the same side was located in the white matter adjacent to the inferior frontal cortex [337 voxels, p = 0.03, peak in MNI (-36, 21, 22.5)]. On the right side, there was only one cluster [4294 voxels, p = 0.02, peak in MNI (27, 46.5, 3)] (Fig 3).

#### Cognitively preserved vs. cognitively impaired patients

There were no areas of significant grey matter volume difference between the cognitively preserved patients and the cognitively impaired patients at P<0.05, corrected. Lowering the threshold to p<0.005 uncorrected did not result in the appearance of any clusters. Substituting non-modulated images in the analysis also failed to reveal any clusters of significant difference.

### Functional imaging findings

Twenty-eight of the healthy controls, 27 of the cognitively preserved patients and 23 of the cognitively impaired patients participated in this part of the study (5 cognitively preserved patients and 5 cognitively impaired patients could not be included because of technical problems with the acquisition and processing of the images; 1 cognitively preserved patient was excluded because of excessive movement). There continued to be no significant differences between the three groups in demographic characteristics, and between the two patient groups in clinical ratings (S1 Table).

#### Behavioural performance

The mean level of performance (d’) on the 1-back and 2-back versions of the n-back task was lower in the cognitively preserved patients than in the healthy controls, and lower in the cognitively impaired patients than in the cognitively preserved patients [1-back: 4.40 (0.57) vs. 4.17 (0.63) vs. 3.67 (1.09); H = 7.56; p = 0.02; 2-back: 3.33 (0.83) vs. 3.00 (0.69) vs. 2.52 (0.73); F = 7.32, p<0.001]. However, only the differences between the controls and the cognitively impaired patients reached significance (S1 Table).

#### Within-group activations and de-activations

In the 2-back vs. baseline comparison the healthy controls showed bilateral activations in the DLFPC, precentral gyri, supplementary motor area, anterior insula, cerebellum, thalamus, basal ganglia, and parts of the temporal and parietal cortex. In the 1-back vs. baseline, activations followed a broadly similar pattern but the clusters were less extensive, the basal ganglia were activated only in the left side and no activations were seen in cerebellum and thalamus (S2 Table, S1 and S2 Figs).

Task-related de-activations in the 2-back vs. baseline contrast were seen bilaterally in the medial frontal cortex, amygdala, hippocampus, the medial parietal cortex, the posterior insula and the lateral parietal cortex. In the 1-back vs. baseline contrast, only the medial frontal cortex showed de-activation (S1 and S2 Figs).

Activations and de-activations in the two groups of euthymic bipolar patients followed a broadly similar pattern to that seen in the controls. However, both the activation and de-activation clusters were noticeably less extensive. The cognitively impaired patients in particular showed less extensive prefrontal activation in 2-back vs. baseline contrast and no de-activation in the medial prefrontal, amygdala, hippocampus and posterior insula in both the 1-back vs. baseline and 2-back vs. baseline contrasts (S1 and S2 Figs).

#### Controls vs. cognitively preserved patients

**Fig 4:**
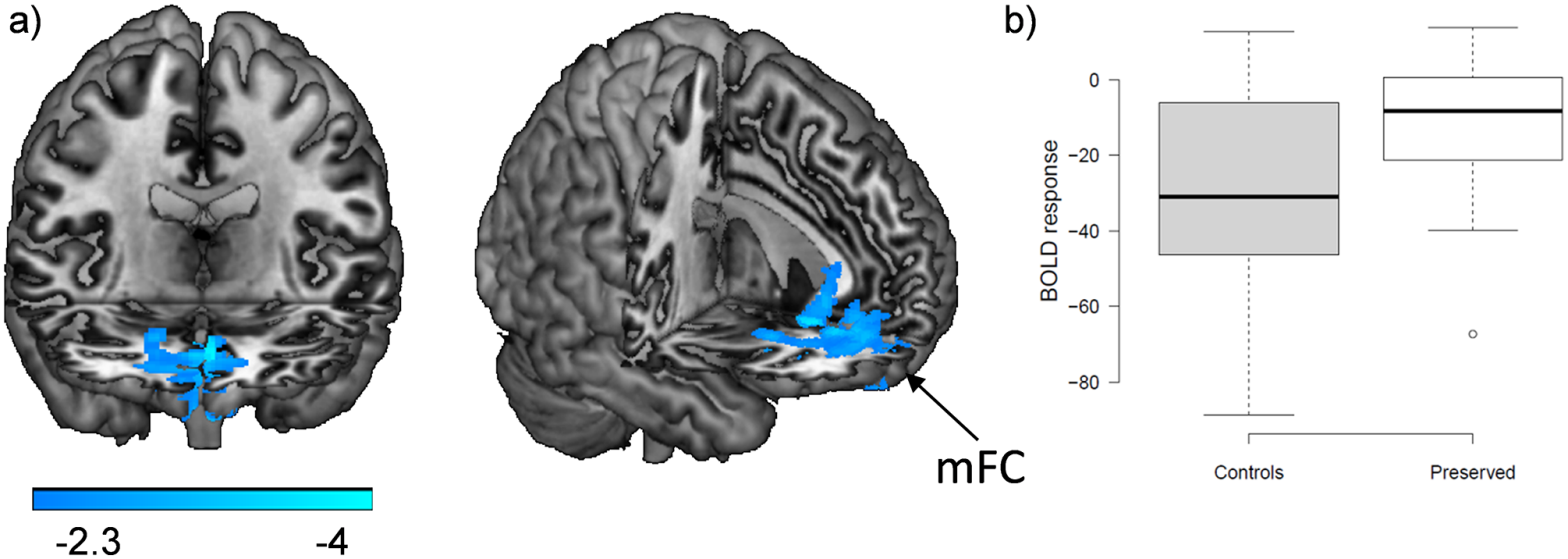
Brain functional changes between controls and cognitively preserved patients. (A) Brain regions where the cognitively preserved patients showed significant failure of de-activation compared with the controls in the 2-back vs. 1-back contrast. MFC: medial frontal cortex. (B) Boxplots of mean de-activations within this ROI.

There were no activation differences between the healthy controls and the cognitively preserved patients in the 1-back vs. baseline or the 2-back vs. baseline contrasts, or in the 2-back vs. 1-back contrast. The cognitively preserved patients did, however, show a cluster of failure of de-activation in comparison to the healthy controls in both contrasts. In the 2-back vs. baseline contrast this cluster was located in the medial prefrontal cortex affecting the gyrus rectus and extended to the medial orbitofrontal and anterior cingulate cortex [4743 voxels, p = 2.18x10^-9^; peak activation in BA11, MNI (4, 34,-8), z score = 4.5]. In the 2-back vs. 1-back contrast the cluster occupied a similar but smaller area in the medial prefrontal cortex [1718 voxels, p = 2.04x10^-4^; peak activation in BA25, MNI (2,36,6), z score = 4.16]. The findings for the 2-back vs. 1-back contrast are shown in Fig 4A. Boxplots of the averaged values in the medial prefrontal region-of-interest (ROI) for the controls and the cognitively preserved patients for this contrast confirm that the differences represented failure of de-activation: the controls showed de-activation whereas in the patients the mean value was close to zero (Fig 4B).

#### Cognitively preserved vs. cognitively impaired patients

**Fig 5:**
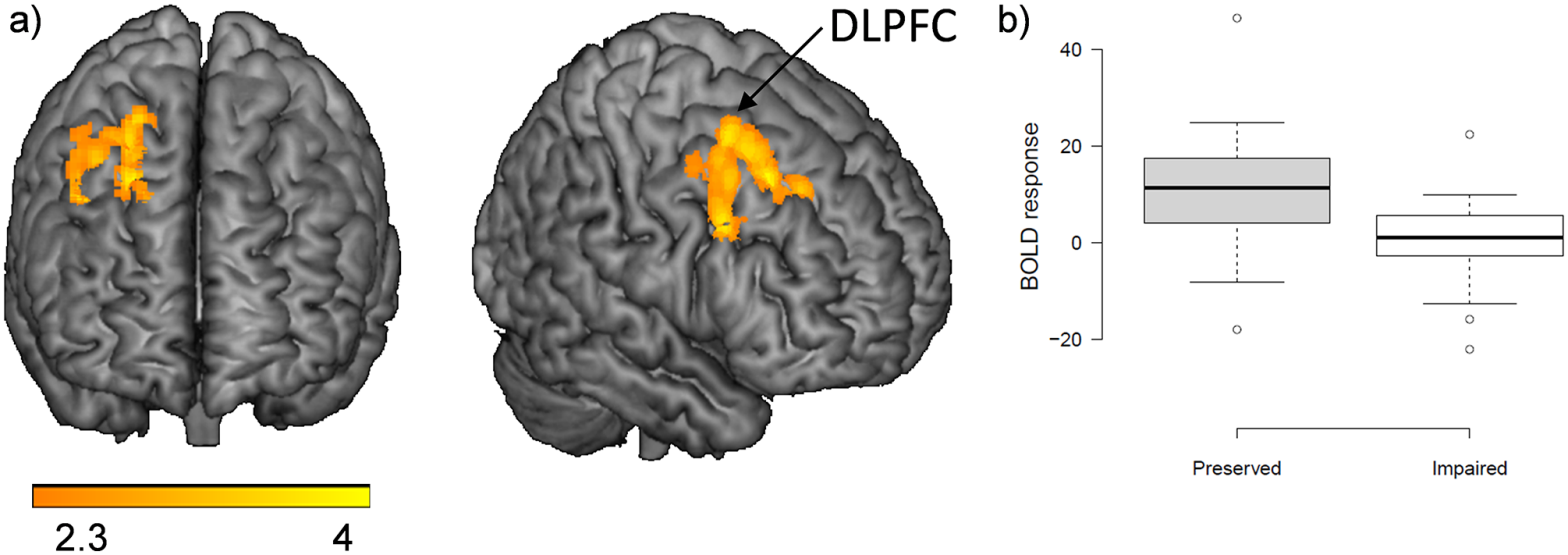
Brain functional changes between cognitively impaired and preserved patients. (A) Brain regions where the cognitively impaired patients showed significantly reduced activation compared with the preserved patients in the 2-back v. 1-back contrast. (B) Boxplots of mean activations within this ROI.

There were no differences between the two patient groups in the 1-back vs. baseline and the 2-back vs. baseline contrasts. The 2-back vs. 1-back contrast, however, revealed a cluster of reduced activation in the cognitively impaired group in the right lateral frontal cortex, extending from the inferior frontal operculum to lateral superior frontal regions and including parts of the DLPFC [905 voxels, *p* = 0.008; peak activation in BA8, right superior frontal, MNI (24, 20, 46), z score = 4.12]. The findings are shown in Fig 5. The two patient groups did not show differences in de-activation.

## Discussion

Cognitive impairment in the euthymic phase—i.e. that is persistent and unrelated to mood disturbance—is now a well-established finding in bipolar disorder. Our study suggests that its basis does not lie in brain structural change. However, there was a positive signal in relation to brain function, with the cognitively impaired patients showing reduced activation in the right DLPFC compared to the cognitively preserved patients.

Given that in neurological disease structural brain damage is commonly associated with neuropsychological deficits, our failure to find differences in grey or white matter volume between bipolar patients with and without cognitive impairment might be considered surprising. One possible reason for this might be that, with sample sizes of 33 and 28 patients, the study might simply have lacked sufficient power to detect differences. Against this, however, is the fact that grey matter changes were still not seen when a more liberal threshold of p<0.005 uncorrected was used in the VBM analysis, or when the modulation step was omitted, which also increases sensitivity [61].

Our negative structural imaging findings might also be considered inconsistent with the current widely held view that the occurrence of persistent cognitive impairment in some patients with bipolar disorder reflects a neurodegenerative process [62]. Of course, being cross-sectional in nature, the study does not speak directly to this issue. Nevertheless, it is interesting to note that the evidence that progressive brain structural change takes place at all in bipolar disorder is actually quite weak. Thus, reviewing the small number of longitudinal studies carried out to date, Lim et al [63] found no evidence for change in whole brain volume over time. Progressive volume reductions were found in the frontal lobe cortex in two small studies (N = 8 and N = 10) but not in a third, larger study (N = 58) which also employed healthy controls. Findings were likewise conflicting for the anterior cingulate cortex, amygdala and hippocampus.

On the other hand, we found evidence that cognitive impairment in euthymic bipolar patients was associated with brain functional changes, specifically reduced activation in a region that conformed reasonably closely to the right DLPFC, although this was only seen in the 2-back vs. 1-back contrast. Our findings here show a notable similarity to those of Oertel-Knöchel et al [33] described in the Introduction—they found reduced activation in the left middle superior frontal gyrus in 26 euthymic bipolar patients, who as a group showed poor memory test performance, during the encoding phase of a memory task (reduced activation was seen in other lateral frontal regions during retrieval). The DLPFC is implicated in both the cognitive, i.e. executive, aspects of frontal lobe function [64] and in long-term memory [65], and so is a plausible location for brain functional changes associated with performance of both types of task in bipolar disorder.

A factor complicating the interpretation of this finding concerns the ‘chicken and egg’ nature of the relationship between cognition and brain activity. Does reduced activation in cognitively impaired euthymic bipolar patients point to underlying regional cerebral dysfunction? Or does it merely index the fact that the patients performed the task more poorly than the cognitively preserved patients and so activated their frontal lobes to a correspondingly lesser degree? To put it another way, would healthy subjects who were below the 5^th^ percentile on a memory or executive test (as some will inevitably be) show less DLPFC activation during n-back performance than those who are above this threshold? This problem has been considered in some depth in the schizophrenia literature e.g. [66–70], where the main conclusion reached has been that there is no simple linear relationship between cognitive performance and regional cortical activation. However, to our knowledge the same issue has not so far been addressed with respect to the cognitive impairment that sometimes accompanies bipolar disorder.

The other functional imaging finding in our study was failure of de-activation in the medial frontal cortex, which was seen in both groups of bipolar patients. This abnormality has been found in several other studies of bipolar disorder [71–73], with one additional study [74] finding failure of de-activation in the posterior cingulate cortex/precuneus. Both the medial frontal cortex and the posterior cingulate cortex/precuneus are components of the default mode network, a series of interconnected brain regions that are active at rest but which de-activate during performance of attention-demanding tasks [75]. Resting state connectivity studies have also implicated the default mode network in bipolar disorder [76]. The function or functions of the default mode network are currently uncertain, although a role in a range of high-level, self-related cognitive operations seems likely [75]. It has also been suggested that the network exerts a general influence on cognitive function—thus, in healthy subjects lower default mode network activity has been found to be associated with more successful task performance, and lapses of attention are associated with reduced de-activation (for a review see [77]). The fact that we found that medial frontal failure of de-activation did not distinguish cognitively preserved from cognitively impaired euthymic patients, suggests that this general modulatory function carried out by the default mode network dysfunction does not play a role in the cognitive impairment seen in euthymic patients with bipolar disorder.

## Conclusions and Limitations

Our findings do not suggest that brain structural alterations are related to the persistent cognitive impairment that is seen in a proportion of patients with bipolar disorder. However, we find evidence that it might be related to functional changes in the prefrontal cortex. Limitations of the study include that our strategy for recruiting patients meant that the cognitively preserved patients were not explicitly matched with the healthy controls for cognitive function, and in fact they were significantly impaired compared to them. Accordingly, this group should be considered to have been only relatively cognitively preserved. Also, the sample sizes in the structural imaging comparison may have been too small to detect subtle volume differences between the two patient groups. Finally, we scanned at 1.5 Tesla, and our examination of white matter was limited to volume measurement only. Use of 3 Tesla scanning and/or examining white matter integrity using DTI might lead to changes related to cognitive impairment in bipolar disorder being found.

## Supporting Information

S1 Dataset Individual demographic, behavioral and fMRI data. (XLSX)

S1 Fig Mean activations and de-activations in the two patient groups and the controls from the 1-back vs. baseline contrast. Areas of significant activations (red- yellow) and deactivations (blue) in the three groups of subjects. The right side of the image is right side of the brain. MNI coordinates for each one of the axial slices are shown in the last row. Colors depict scores from statistical z maps (negative values in the deactivations). A: Healthy controls; B: Cognitively preserved patients; C: Cognitively impaired patients.
(TIFF)

S2 Fig Mean activations and de-activations in the two patient groups and the controls from the 2-back vs. baseline contrast. Areas of significant activations (red- yellow) and deactivations (blue) in the three groups of subjects. The right side of the image is right side of the brain. MNI coordinates for each one of the axial slices are shown in the last row. Colors depict scores from statistical z maps (negative values in the deactivations). A: Healthy controls; B: Cognitively preserved patients; C: Cognitively impaired patients.
(TIFF)

S1 Table Demographic and psychopathological characteristics in the fMRI sample (SDs in brackets). (DOCX)

S2 Table Clusters of significant activation/de-activation in the comparison between the 1-back vs. baseline, 2-back vs. baseline and between the 1-back vs. 2-back contrasts. (DOCX)
