# Supplementary figures and images for: Structural and Functional Brain Correlates of Cognitive Impairment in Euthymic Patients with Bipolar Disorder
Source: PLoS One. 2016 Jul 22;11(7):e0158867. doi: 10.1371/journal.pone.0158867 (PMC4957815; doi:10.1371/journal.pone.0158867)

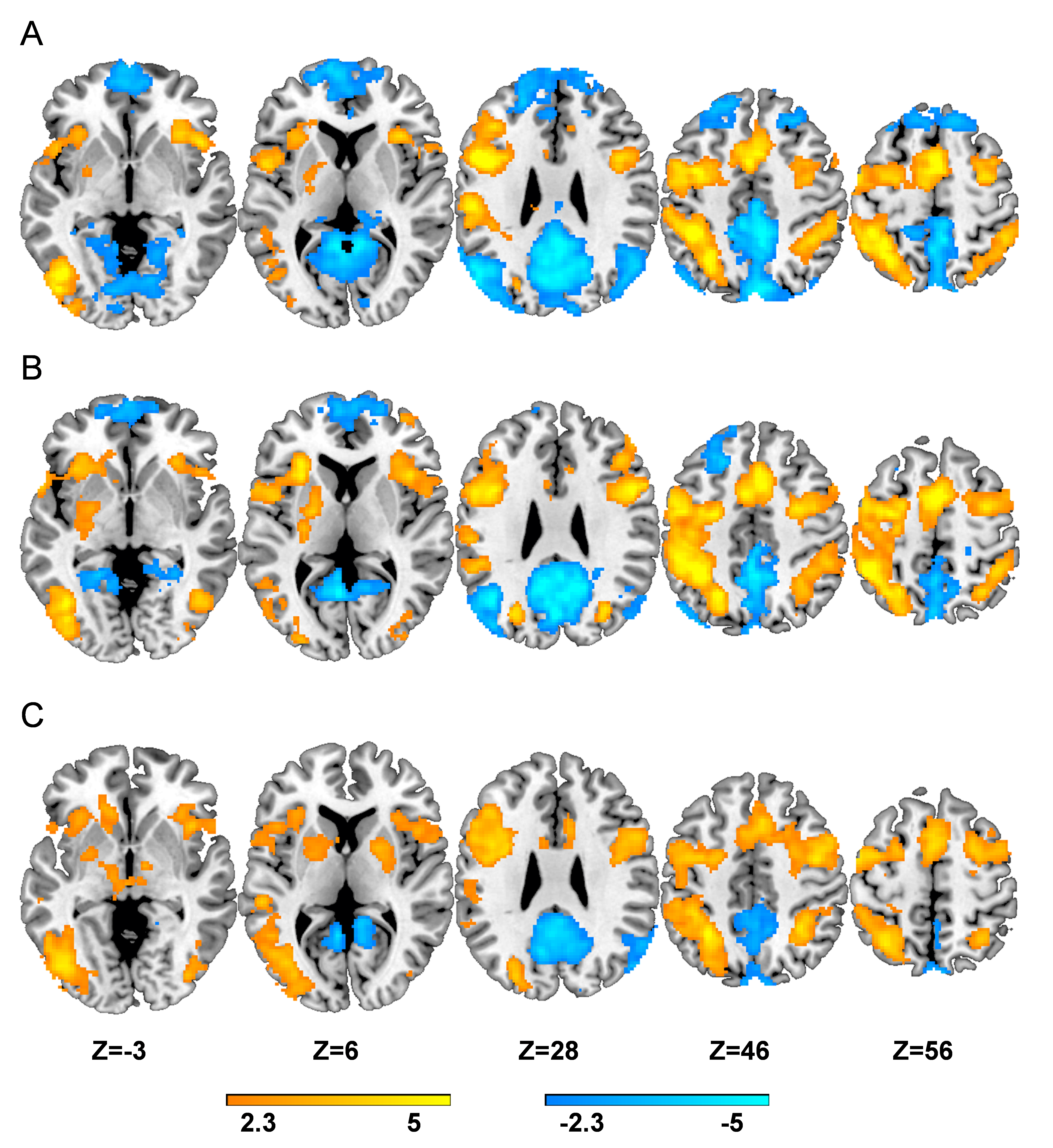

Supplement: S1 Fig — Areas of significant activations (red- yellow) and deactivations (blue) in the three groups of subjects. The right side of the image is right side of the brain. MNI coordinates for each one of the axial slices are shown in the last row. Colors depict scores from statistical z maps (negative values in the deactivations). A: Healthy controls; B: Cognitively preserved patients; C: Cognitively impaired patients. (TIFF) [file pone.0158867.s002.tiff]

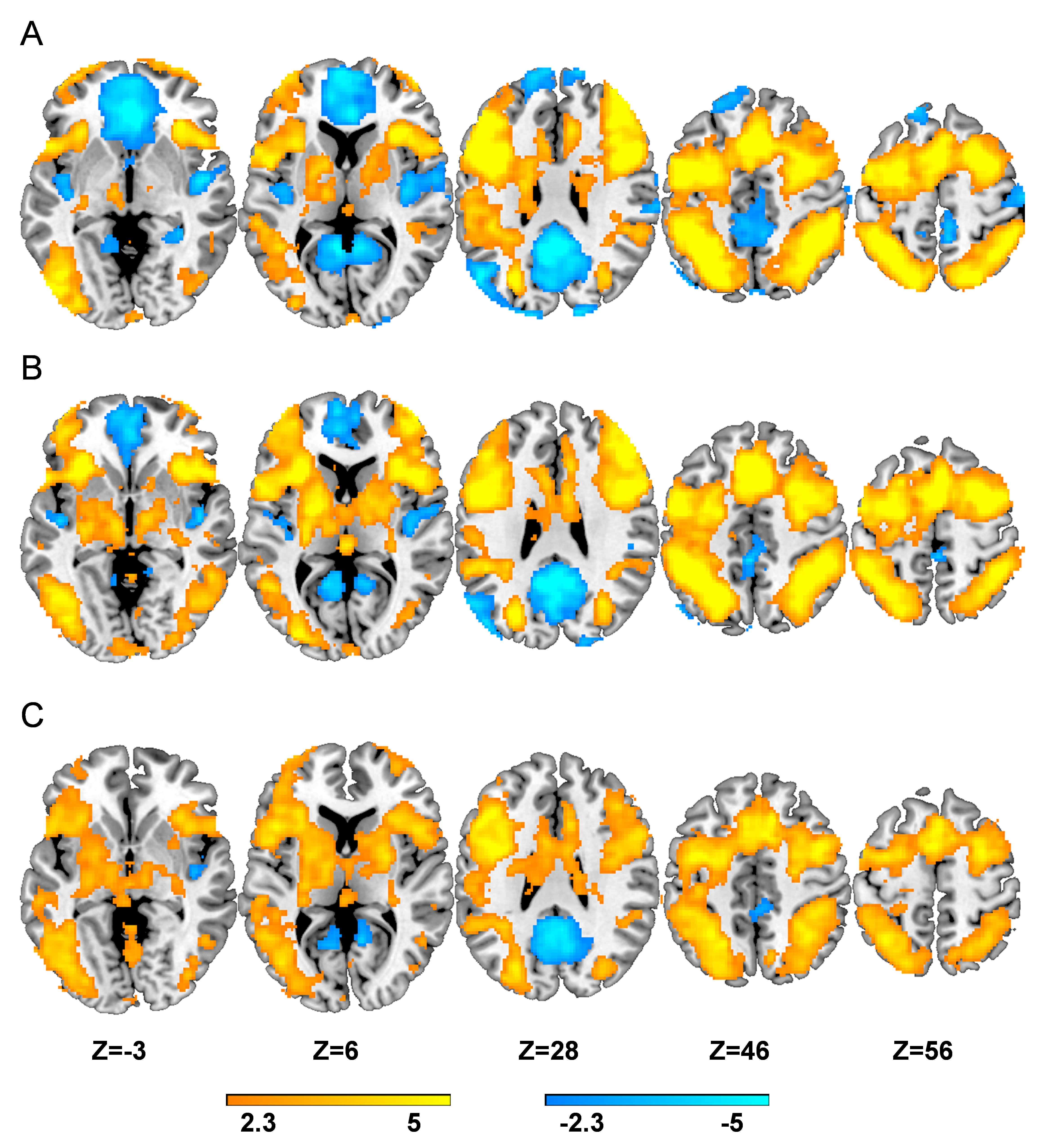

Supplement: S2 Fig — Areas of significant activations (red- yellow) and deactivations (blue) in the three groups of subjects. The right side of the image is right side of the brain. MNI coordinates for each one of the axial slices are shown in the last row. Colors depict scores from statistical z maps (negative values in the deactivations). A: Healthy controls; B: Cognitively preserved patients; C: Cognitively impaired patients. (TIFF) [file pone.0158867.s003.tiff]
