# Supplementary material for: Structural and Functional Brain Correlates of Cognitive Impairment in Euthymic Patients with Bipolar Disorder
Source: PLoS One. 2016 Jul 22;11(7):e0158867. doi: 10.1371/journal.pone.0158867 (PMC4957815; doi:10.1371/journal.pone.0158867)
Supplement: S1 Table — (DOCX) [file pone.0158867.s004.docx]

**S1 Table:** Demographic and psychopathological characteristics in the fMRI sample (SDs in brackets).

|  | Controls (n=28) | Cognitively preserved (n=27) | Cognitively impaired (n=23) | Statistics | Post hoc testing |
| --- | --- | --- | --- | --- | --- |
| Age | 44.01 (6.03) | 44.49 (6.99) | 46.50 (7.82) | F=0.89 p=0.41 |  |
| Sex (M/F) | 12/16 | 15/12 | 13/10 | χ^2^=1.25 p=0.54 |  |
| Estimated premorbid IQ (TAP) | 105.93 (7.25) | 106.70 (5.59) | 103.52 (8.97) | H=1.75 p=0.42 |  |
| BADS profile score | 19.18 (2.40) | 17.33 (2.11) | 13.91 (3.50) | F=24.57 p<0.001 | CI < CP (p<0.001) |
|  |  |  |  |  | CI < CON (p<0.001) |
|  |  |  |  |  | CP < CON (p=0.04) |
| RBMT screening score | 10.61 (1.64) | 9.93 (1.47) | 6.17 (1.30) | H=45.99 p<0.001 | CI < CP (p<0.001) |
|  |  |  |  |  | CI < CON (p<0.001) |
| Duration of illness (years) |  | 16.60 (7.19) | 18.98 (8.64) | t=1.06 p=0.29 |  |
| YMRS score |  | 1.33 (1.90) | 1.95 (2.17) | U=247.50 p=0.29 |  |
| HRSD score |  | 2.44 (2.10) | 2.41 (2.38) | U=281.5 p=0.75 |  |
| GAF score |  | 79.76 (11.99) | 75.52 (12.57) | t=-1.12 p=0.27 |  |
| D’ 1-back | 4.40 (0.57) | 4.17 (0.63) | 3.67 (1.09) | H=7.56 p=0.02 | CI < HC (p=0. 01) |
| D’ 2-back | 3.33 (0.83) | 3.00 (0.69) | 2.52 (0.73) | F=7.32 p<0.001 | CI < HC (p=0.001) |

IQ, intelligence quotient; TAP, Word Accentuation Test; BADS, Behavioural Assessment of the Dysexecutive Syndrome; RBMT, Rivermead Behavioural Memory Test; YMRS, Young Mania Rating Scale; HRSD, Hamilton Rating Scale for Depression; GAF, Global Assessment of Functioning; F, one-way ANOVA test; χ^2^, Chi-square test; H, one-way Kruskal-Wallis test; U, Mann-Whitney test; CON, controls; CP, cognitively preserved; CI, cognitively impaired.
