# Supplementary material for: Structural and Functional Brain Correlates of Cognitive Impairment in Euthymic Patients with Bipolar Disorder
Source: PLoS One. 2016 Jul 22;11(7):e0158867. doi: 10.1371/journal.pone.0158867 (PMC4957815; doi:10.1371/journal.pone.0158867)
Supplement: S2 Table — (DOCX) [file pone.0158867.s005.docx]

**S2 Table:**  Clusters of significant activation/de-activation in the comparison between the 1-back vs baseline, 2-back vs. baseline and between the 1-back vs 2-back contrasts.

|  | Cluster | | | Local Maxima | |
| --- | --- | --- | --- | --- | --- |
|  | Brodmann areas | Voxels | P value | Z value | MNI x,y,x |
| **1-back vs baseline** |  |  |  |  |  |
| **Healthy controls** |  |  |  |  |  |
| ***Activations*** |  |  |  |  |  |
| Frontal inferior operculum left | 44 | 15238 | p<0.001 | 6.55 | -50,8,28 |
| Insula right | 48 | 2508 | p<0.001 | 4.69 | 36,22,4 |
| Parietal inferior right | 40 | 1549 | p<0.001 | 4.73 | 44,-44,52 |
| ***De-activations*** |  |  |  |  |  |
| Cuneus right | 7 | 20905 | p<0.001 | 5.7 | 6,-76,40 |
| Frontal medial orbital right | 10 | 7064 | p<0.001 | 5.06 | 2,66,-8 |
| Temporal middle right | 21 | 846 | p=0.003 | 3.97 | 60,-10,-16 |
| **Cognitively preserved** |  |  |  |  |  |
| ***Activations*** |  |  |  |  |  |
| Parietal inferior left | 40 | 12177 | p<0.001 | 6.43 | -32,-48,42 |
| Supplementary motor area right | 6 | 7196 | p<0.001 | 6.03 | 2,4,58 |
| Occipital inferior left | 19 | 2254 | p<0.001 | 5.66 | -42,-66,-8 |
| Angular right | 40 | 2051 | p<0.001 | 4.75 | 30,-52,42 |
| Temporal inferior right | 19 | 1416 | p<0.001 | 4.99 | 46,-68,-10 |
| Cerebelum 4-5 right | 18 | 1003 | p<0.001 | 4.01 | 8,-52,-16 |
| Pallidum left | - | 743 | p=0.006 | 4.19 | -24,-6,4 |
| ***De-activations*** |  |  |  |  |  |
| Precuneus right | 23 | 12016 | p<0.001 | 5.9 | 10,-56,22 |
| Frontal medial orbital right | 10 | 1673 | p<0.001 | 4.36 | 4,64,0 |
| Frontal superior left | 9 | 836 | p=0.003 | 4.33 | -20,26,44 |
| Temporal middle right | 39 | 651 | p=0.01 | 3.98 | 60,-64,18 |
| **Cognitively impaired** |  |  |  |  |  |
| ***Activations*** |  |  |  |  |  |
| Parietal inferior left | 7 | 18768 | p<0.001 | 5.26 | -30,-54,44 |
| Cingulum Middle right | 32 | 8350 | p<0.001 | 5.18 | 10,16,40 |
| Parietal inferior right | 40 | 981 | p=0.001 | 4.7 | 32,-48,48 |
| ***De-activations*** |  |  |  |  |  |
| Precuneus right | 23 | 5707 | p<0.001 | 4.91 | 8,-52,32 |
| Temporal middle right | 22 | 874 | p=0.003 | 4.5 | 60,-60,20 |
| **2-back vs baseline** |  |  |  |  |  |
| **Healthy controls** |  |  |  |  |  |
| ***Activations*** |  |  |  |  |  |
| Frontal inferior operculum left | 44 | 69990 | p<0.001 | 7.77 | -52,10,24 |
| ***De-activations*** |  |  |  |  |  |
| Frontal medial orbital left | 11 | 21662 | p<0.001 | 6.63 | -2,40,-8 |
| Precuneus right | 30 | 9373 | p<0.001 | 5.81 | 2,-50,26 |
| Postcentral right | 3 | 1648 | p<0.001 | 4.87 | 28,-46,76 |
| **Cognitively preserved** |  |  |  |  |  |
| ***Activations*** |  |  |  |  |  |
| Parietal inferior left | 40 | 74650 | p<0.001 | 7.58 | -42,-44,42 |
| ***De-activations*** |  |  |  |  |  |
| Precuneus left | 23 | 5320 | p<0.001 | 6.58 | -4,-56,22 |
| Frontal medial orbital left | 11 | 2776 | p<0.001 | 4.95 | -2,54,-10 |
| Temporal pole superior left | 38 | 2456 | p<0.001 | 5.07 | -32,8,-24 |
| Temporal pole superior right | 38 | 1750 | p<0.001 | 4.42 | 30,10,-20 |
| Angular left | 39 | 1095 | p=0.001 | 4.66 | -52, -68,28 |
| Postcentral right | 3 | 794 | p=0.009 | 4.37 | 26,-40,76 |
| Temporal superior right | 48 | 592 | p=0.04 | 4.47 | 46,-10,0 |
| **Cognitively impaired** |  |  |  |  |  |
| ***Activations*** |  |  |  |  |  |
| Cerebelum crus2 right | - | 65970 | p<0.001 | 6.04 | 10,-80,-28 |
| ***De-activations*** |  |  |  |  |  |
| Precuneus left | 23 | 3125 | p<0.001 | 4.89 | -4,-54,26 |
| Temporal inferior right | 20 | 620 | p=0.02 | 4.2 | 50,-6,-36 |
| Postcentral right | 3 | 595 | p=0.03 | 3.9 | 28,-38,74 |
| **2-back vs 1-back** |  |  |  |  |  |
| **Healthy controls** |  |  |  |  |  |
| ***Activations*** |  |  |  |  |  |
| Cerebelum crus 1 left | - | 81503 | p<0.001 | 6.98 | -34,-62,-32 |
| ***De-activations*** |  |  |  |  |  |
| Cingulum anterior left | 11 | 17009 | p<0.001 | 6.01 | -2,34,-8 |
| Postcentral left | 4 | 902 | p=0.003 | 4.66 | -54,-16,56 |
| Postcentral right | 3 | 868 | p=0.004 | 4.32 | 32,-36,74 |
| **Cognitively preserved** |  |  |  |  |  |
| ***Activations*** |  |  |  |  |  |
| Parietal superior left | 7 | 59851 | p<0.001 | 6.28 | -26,-58,50 |
| ***De-activations*** |  |  |  |  |  |
| Temporal pole superior left | 38 | 4375 | p<0.001 | 4.96 | -32,16,-30 |
| Temporal pole superior right | 38 | 1932 | p<0.001 | 4.57 | 26,12,-26 |
| Precentral right | 4 | 625 | p=0.04 | 4.04 | 40,-22,70 |
| **Cognitively impaired** |  |  |  |  |  |
| ***Activations*** |  |  |  |  |  |
| Cerebelum crus1 left | 37 | 27654 | p<0.001 | 4.77 | -42,-58,-24 |
| Precentral right | 6 | 5109 | p<0.001 | 4.54 | 30,-4,52 |
| Frontal superior left | 10 | 5084 | p<0.001 | 4.4 | -14,72,4 |
| ***De-activations*** |  |  |  |  |  |
| Cingulum anterior left | 11 | 1581 | p<0.001 | 3.53 | -6,32,-2 |
| Fusiform right | 20 | 879 | p<0.001 | 4.56 | 32,0,-42 |
